# Effect of targeted screening and decolonisation for methicillin-sensitive Staphylococcus aureus (MSSA) in elective hip and knee arthroplasty

**DOI:** 10.3205/id000107

**Published:** 2026-06-02

**Authors:** Jana Schroeder, Irit Nachtigall, Martin Wolke, Cathrin Kodde

**Affiliations:** 1 Department of Hospital Hygiene and Microbiology, Stiftung Mathias-Spital, Rheine, Germany; 2 Institute for Hygiene and Environmental Medicine, Vivantes Medical School Berlin, Germany; 3 Department of Infectious Diseases and Respiratory Medicine, Charité – University Medicine Berlin, Germany

## Abstract

**Background:**

Nasal and pharyngeal colonization with *Staphylococcus aureus* (*S. aureus*) is a major risk factor for postoperative wound infections, particularly following orthopedic procedures. Preoperative targeted decolonisation reduces surgical site infections (SSIs) and is recommended by several national and international guidelines. However, real-world data on the sustained effectiveness of standardized targeted decolonisation programs in orthopedic surgery remain limited.

**Objective:**

To evaluate the long-term effectiveness of a standardized preoperative *S. aure*us screening and targeted octenidine-based decolonisation protocol in reducing postoperative wound infections among orthopedic patients in a secondary care hospital.

**Methods:**

In this retrospective single-center interventional cohort study, all patients undergoing elective hip and knee replacement surgery between 2016 and 2023 were screened preoperatively for *S. aureus* using nasal and throat swabs. Patients testing positive underwent decolonisation with octenidine-based antiseptics. Postoperative infection rates were compared between a pre-intervention period (January 2016 to August 2021) and a post-intervention period (January 2022 to December 2023). A predefined implementation phase (September to December 2021), during which routine screening and decolonisation procedures were established, was excluded from comparative analyses.

**Results:**

Among 436 and 511 screened patients prior to elective hip- and knee replacement in 2022 and 2023, respectively, *S. aureus* carriage was detected in 18%. Implementation of the targeted decolonisation protocol reduced *S. aureus*-associated wound infections in our reference hospital from 2.89–0.99 per 100 surgeries during the pre-intervention period (2016–2021) to 0.45–0.00 in 2022 and 2023, respectively.

**Conclusion:**

Routine preoperative *S. aureus* screening followed by targeted octenidine-based decolonisation significantly and sustainably reduced postoperative infection rates. These findings provide robust real-world evidence supporting the integration of standardized *S. aureus* decolonisation protocols into routine orthopedic practice.

## Introduction

Surgical Site Infections (SSI) are potentially life-threatening complications among patients undergoing orthopedic surgery and are associated with substantial morbidity, prolonged hospitalization, and increased healthcare costs. They represent one of the most frequent healthcare-associated infections in surgical patients and may result in implant failure or the need for revision surgery. *Staphylococcus aureus* (*S. aureus*) is the most common pathogen responsible for SSIs in orthopedic patients. Other frequently isolated organisms include coagulase-negative staphylococci (particularly *Staphylococcus epidermidis*), Enterococcus species, *Escherichia coli*, *Pseudomonas aeruginosa*, and Enterobacterales species. These pathogens reflect both endogenous flora and environmental or device-associated sources, highlighting the multifactorial nature of SSI pathogenesis in orthopedic surgery. Humans represent the primary reservoir for *S. aureus*, although animal reservoirs exist as well. Given the central role of human colonization as a source of subsequent infection, screening for nasal *S. aureus* carriage represents a preventive strategy in elective arthroplasty. Targeted methicillin-sensitive *S. aureus* (MSSA) decolonisation in elective hip and knee replacement surgery involves preoperative screening for *S. aureus* nasal carriage, followed by decolonisation of identified carriers. The Infectious Diseases Society of America and the Kommission für Krankenhaushygiene und Infektionsprävention (KRINKO) in Germany recommend this approach as an essential practice to prevent SSI in acute-care hospitals, particularly for procedures involving hardware implantation such as joint arthroplasty [1], [2]. Universal and targeted screening and decolonisation represent two distinct strategies for preventing *S. aureus*-associated surgical SSI. Universal decolonisation involves treating all patients undergoing a specific procedure without prior identification of colonisation status. This approach avoids logistical challenges associated with screening and ensures treatment of all carriers. In orthopedic surgery, universal strategies have demonstrated reductions in *S. aureus*-related SSI, particularly in high-risk settings. However, as only a minority of patients are MSSA carriers, universal decolonisation results in substantial overtreatment, increased antiseptic exposure and potential selective pressure, raising concerns regarding antimicrobial stewardship and microbiome disruption. Targeted screening and decolonisation limits treatment to patients with proven *S. aureus* colonisation, identified preoperatively by nasal/throat screening. This strategy is well suited to elective hip and knee arthroplasty, where sufficient time for screening and decolonisation is available. Targeted approaches have been shown to effectively reduce SSI and periprosthetic joint infection while minimising unnecessary antiseptic use. In addition, targeted screening supports risk stratification and aligns with stewardship principles. Although targeted strategies require reliable diagnostics and timely workflows, their selective nature and favourable benefit-risk profile make them particularly appropriate for elective arthroplasty procedures. Overall, targeted screening and decolonisation offer an effective and resource-efficient alternative to universal approaches in *S. aureus* prevention. 

Colonization mostly occurs in the nasopharynx, with carriage rates ranging from 15% and 40% in healthy adults [3]. Individuals working in agriculture, particularly in pig-fattening farms, are at increased risk of exposure and colonization due to frequent contact with livestock, which can serve as a reservoir of *S. aureus*. Known patient-related risk factors include obesity, diabetes mellitus, and malnutrition, all of which are associated with impaired wound healing and higher SSI rates [4], [5]. In addition, preoperative lifestyle modifications may contribute to infection reduction. Numerous studies have demonstrated that *S. aureus* colonization markedly increases the risk of postoperative wound infections [6], [7], [8], [9]. Consequently, prevention of SSIs in orthopedic surgery relies on a multifactorial perioperative strategy combining strict asepsis, optimized antimicrobial prophylaxis, and targeted screening and decolonisation measures. Several studies have shown that preoperative nasal screening and subsequent decolonisation of *S. aureus* carriers significantly reduce postoperative infection rates in orthopedic patients [10], [11]. Standard decolonisation protocols typically include intranasal mupirocin and chlorhexidine body washes, both of which have been shown to decrease SSIs compared with unscreened populations [12], [13]. These findings have been incorporated into several national and international guidelines recommending *S. aureus* screening and decolonisation prior to high-risk procedures such as cardiothoracic and orthopedic surgery [2], [14], [15]. However, data on the effectiveness of alternative antiseptic agents and broader decolonisation approaches in orthopedic populations remain limited. 

The present study aimed to evaluate whether the current targeted decolonisation protocol using octenidine, applied intranasally, intraorally, and for whole-body washing effectively achieves *S. aureus* decolonisation and reduces postoperative wound infections in orthopedic patients. 

## Methods

A retrospective, single-center interventional cohort study was conducted to evaluate the efficacy of a targeted *S. aureus* decolonisation protocol in hospitalized patients prior to elective knee and hip replacement surgery and its impact on *S. aureus* infection rates. The study was carried out at a hospital with 25 beds affiliated with the Mathias Foundation (Stiftung Mathias-Spital), a charitable healthcare organization in Germany operating multiple hospitals and care facilities. Within this network, a hospital primarily specialized in elective hip and knee implant surgery was selected. In January 2018, a medically led Institute for Hospital Hygiene and Microbiology was established at this site. From September 2021 onward, preoperative *S. aureus* screening (nasal and throat swabs) was implemented in accordance with the 2018 KRINKO recommendations for the ‘Reduction of postoperative wound infections’ [2]. This period was considered an implementation phase and was not included in the comparative outcome analyses. A postoperative wound infection was defined as a surgical site infection occurring at the incision site after an operative procedure, involving only the skin and/or subcutaneous tissue. The first signs or symptoms of infection had to occur within 30 days after surgery and at least one of the following criteria had to be met: purulent drainage from the superficial incision; isolation of a pathogen from an aseptically obtained specimen from the incision or subcutaneous tissue; or the presence of at least one local sign of infection – such as pain or tenderness, localized swelling, erythema, or increased local warmth – together with deliberate opening of the incision. The diagnosis of a wound infection had to be made by the treating physician [16]. The primary endpoint was the detection of *methicillin-susceptible* *S. aureus* (MSSA) or *methicillin-resistant S. aureus* (MRSA) in wound infection following hip or knee arthroplasty. The pre-interventional period was defined as January 2016 to August 2021, during which no systematic preoperative *S. aureus* screening was performed. The interventional period extended from September 2021 to December 2021, reflecting the stepwise introduction and establishment of routine screening and decolonisation procedures. The post-interventional period was defined as January 2022 to December 2023, during which the intervention was fully implemented. Statistical analysis was performed using Fisher’s exact test (two-sided, α=0.05). Effect measures included relative risk (RR) with 95% confidence intervals (Katz method), absolute risk reduction (ARR), and number needed to treat (NNT=1/ARR). The denominators were the number of operated patients in each phase (n=4,055). The effect of preoperative *S. aureus* screening and subsequent decolonisation on postoperative wound infection rates was analysed retrospectively and anonymously using routine clinical data collected between 2016 and 2023. Two patient cohorts were analysed: a historical control group (2016–2020) without systematic screening or decolonisation, and an intervention group (2022–2023) in which all consecutive patients underwent preoperative screening and targeted decolonisation when *S. aureus* was detected, ensuring complete cohort inclusion. Patients were screened for MSSA and MRSA using nasal and throat swabs obtained during the first preoperative contact, no later than seven days before surgery. Nasal and throat samples were collected using commercially available sterile cotton swabs and processed in an external microbiology laboratory using chromogenic agar on half petri dishes for the identification and differentiation of *S. aureus*. The primary outcome was the rate of *S. aureus* wound infections, expressed as infections per 100 surgeries, comparing baseline data from 2016 with data from 2023. The following decolonisation protocol was used: patients testing positive for *S. aureus* were instructed to perform a 5-day preoperative decolonisation regimen consisting of daily showers using octenisan^®^ washing lotion (1-minute contact time) with daily towel and laundry changes. In addition, patients used a chlorhexidine mouthwash solution (15 ml for 20 seconds, twice daily) and applied octenisan^®^ nasal gel three times daily. If MRSA was detected, patients were referred to the local public health authority in Lower Saxony or to their primary care physician in North Rhine-Westphalia for MRSA decolonisation. The standard MRSA-decolonisation did not differ from the MSSA decolonisation, but 14 days after completion of the protocol, three control nasal-/throat-swabs were recommended for these patients. Postoperative wound infections were assessed within 30 days after surgery according to the KISS (Hospital Infection Surveillance System) definition by the attending physician. 

The Ethics Committee of the Ärztekammer Westfalen-Lippe determined that formal ethics approval was not required for this project (file number 2025-240-f-N).

## Results

Between 2016 and 2023, a total of 4,055 elective primary hip and knee arthroplasties were performed at the study site. Of these, 3,102 procedures (76.5%) were conducted during the pre-intervention period (2016–2021), which served as the historical control group, and 953 procedures (23.5%) during the post-intervention period (2022–2023).

Overall, 1,561 male (38.5%) and 2,494 female patients (61.5%) underwent surgery. The proportion of male patients varied slightly across the study years, ranging from 34.6% in 2022 to 40.8% in 2023, without a consistent temporal trend. The median age of the overall cohort ranged between 66 and 71 years across the study period. 

**Figure 1:**
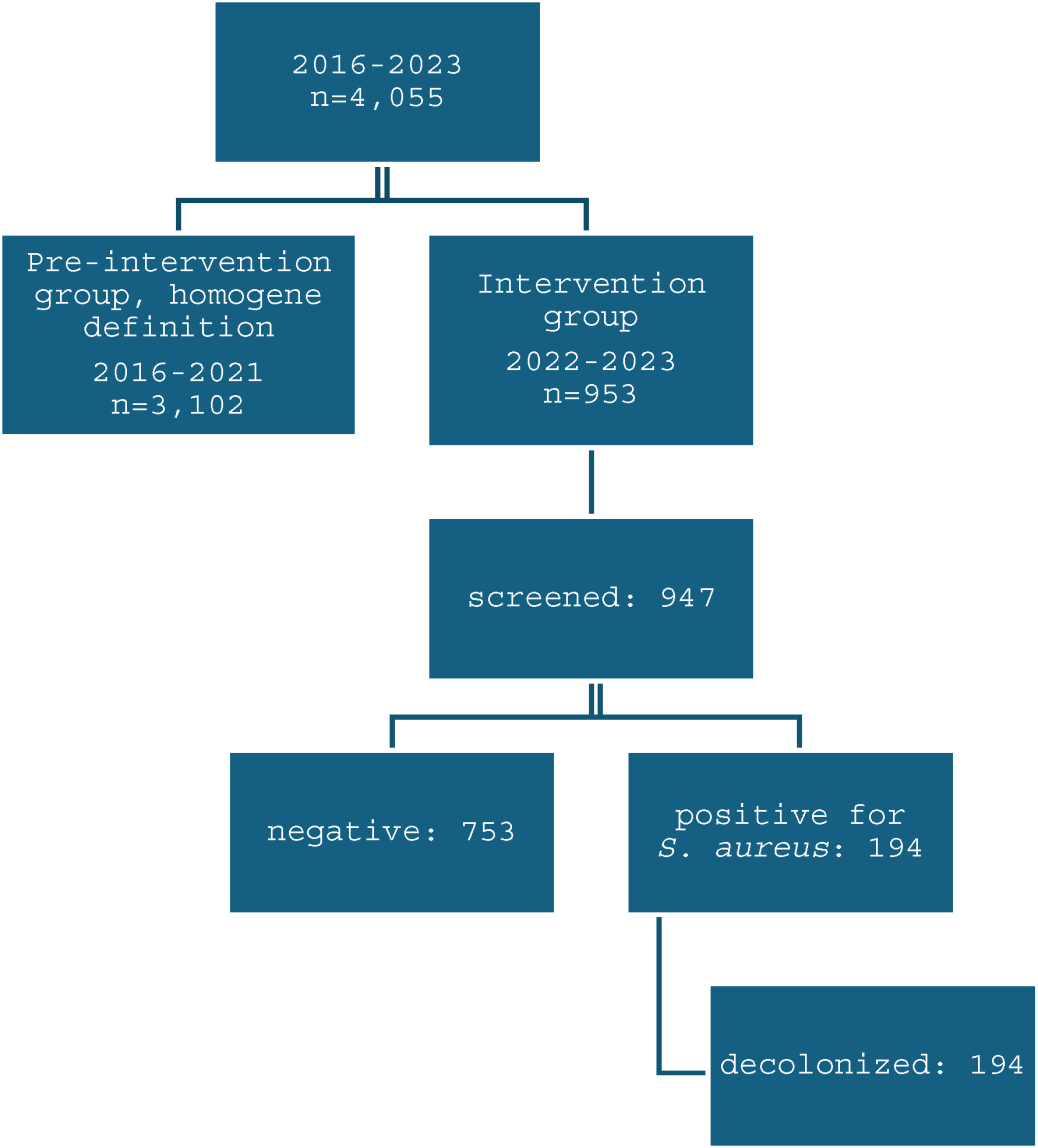
Course of screening measures and detection rates of *S. aureus* (MSSA/MRSA) 2016–2023

With regard to comorbidities, diabetes mellitus was the most frequent underlying condition, present in 567 of 4,055 cases (14.0%, see Figure 1 (Fig. 1)). The annual proportion of patients with diabetes ranged from 13.5% (84/622) in 2016 to 20.0% (104/514) in 2023. Chronic renal insufficiency was documented in 112 cases (2.8%) and heart failure in 95 cases (2.3%) over the entire study period. The yearly proportion of renal insufficiency ranged from 2.0% to 6.6%, and that of heart failure from 1.6% to 4.9%, without a clear increasing or decreasing trend over time. Overall, baseline demographic characteristics and comorbidity profiles remained broadly comparable throughout the study period (Table 1 (Tab. 1)).

Until 2021, targeted MRSA screening based on individual risk factors was performed, with the number of screening tests declining from 551 in 2016 to 156 in 2021 (see Table 2 (Tab. 2)). Following the implementation of systematic combined screening, isolated MRSA screening was discontinued. During the implementation phase in 2021, 198 combined screenings were conducted, increasing to 436 in 2022 and 511 in 2023. The proportion of positive *S. aureus* screenings remained stable at approximately 18–21% during the intervention period. Concurrently with the introduction of systematic screening and decolonisation, a marked reduction in postoperative wound swabs was observed, decreasing from 141 in 2016 to 45 in 2023 (68.1%). The incidence of *S. aureus*-positive wound findings per 100 procedures declined from 2.89 in 2016 to 0.00 in 2023, with intermediate fluctuations over time. Across the entire study period, 51 *S. aureus* wound isolates were identified, comprising 41 MSSA and 10 MRSA isolates. MRSA was detected in wound cultures between 2016 and 2019 but was no longer observed from 2020 onward. MSSA remained the predominant pathogen but showed a substantial decline following implementation of systematic screening and decolonisation. In the historical cohort (2016–2021), 39 MSSA wound infections were documented, compared to only two cases in the post-intervention period (2022–2023), with no infections observed in 2023.

## Discussion

The main finding of this study was a marked reduction in *S. aureus* wound infection rates from 2.89 per 100 procedures in 2016 to zero in 2023. This substantial decline was associated with the implementation of a standardized screening and targeted decolonisation protocol. 

The rationale for such strategies has been established since the first description of targeted decolonisation by Bode et al. [17] and is supported by meta-analyses and randomized controlled trials demonstrating a significant reduction in postoperative *S. aureus* SSI. 

In our study, we deliberately implemented a targeted approach to minimize unnecessary topical antibiotic exposure and mitigate resistance development. The protocol comprised preoperative screening followed by octenidine-based intranasal treatment combined with antiseptic whole-body washes for five consecutive days. While universal decolonisation without screening may be easier to implement and potentially more cost-effective, targeted screening offers the advantage of enabling tailored perioperative antibiotic prophylaxis based on carriage status. Both strategies have demonstrated efficacy in reducing *S. aureus* SSIs in elective hip and knee arthroplasty [1], [18]. Rapid identification and decolonisation of carriers has been associated with substantial reductions in overall and deep-incisional *S. aureus* SSIs, as well as decreased one-year mortality [1]. Previous studies have predominantly evaluated mupirocin-based regimens, frequently in the context of universal rather than targeted strategies. 

All patients received standardized perioperative prophylaxis with a single dose of cefazolin in addition to the targeted decolonisation protocol. The protocol was feasible in routine clinical practice and was associated with a marked reduction in SSI rates. Our findings align with a growing body of evidence demonstrating the effectiveness of preoperative screening and decolonisation across various orthopedic populations, including elective joint replacement [10], elective spinal surgery [19], and orthopedic trauma patients [20]. 

*S. aureus* colonization is a well-established risk factor for postoperative infection, particularly in surgical patients. Nasal carriage plays a central role in the pathogenesis of invasive disease, and eradication of carriage has been shown to reduce infection rates in high-risk populations. These observations provide the biological rationale for preoperative screening and targeted decolonisation strategies in elective orthopedic surgery. Elimination of *S. aureus* carriage represents a biologically plausible and evidence-based preventive strategy in surgical patients. 

In a representative interventional study, *S. aureus*-associated prosthetic joint infections were reduced from 66.6% of all PJIs prior to intervention to a single case during the intervention period (odds ratio 0.15; 95% CI 0.004–0.94) [20]. Similarly, Chen et al. [21], Romero-Palacios et al. [22], and Portais et al. [23] demonstrated significant reductions in colonization and infection rates following structured decolonisation protocols. Additional institutional analyses reported decreases in MSSA-related PJI rates from 0.75% to 0.25% and reductions in overall PJI rates from 1.92% to 1.41% [24].

The magnitude of reduction observed in our cohort (96.4%) appears higher than that reported in several previous institutional studies. This may reflect consistent interdisciplinary implementation, high adherence, and the combined use of intranasal octenidine with antiseptic body washes. The avoidance of mupirocin may further enhance long-term sustainability by reducing selective pressure for resistance.

Regarding antiseptic agents, chlorhexidine is widely used and effective against MSSA but has been associated with reduced susceptibility in some *S. aureus* strains and rare yet severe hypersensitivity reactions. Octenidine dihydrochloride exhibits broad antimicrobial activity, favorable tolerability, and minimal systemic absorption, with a low reported risk of clinically relevant resistance. Current evidence suggests non-inferiority of octenidine compared with chlorhexidine in decolonisation protocols, and it may offer practical advantages in repeated or targeted strategies. An additional observation in our cohort was the absence of MRSA in wound cultures after 2021, which may reflect a secondary effect of the screening and decolonisation strategy. However, given the observational design and extended study period, causality cannot be attributed solely to the intervention. The sustained decline in SSI rates over eight years likely reflects both the implemented protocol and concurrent institutional developments. Several structural and organizational changes may have acted as confounders, including certification as an endoprosthetics center March 2015 increased standardization of perioperative workflows, enhanced infection prevention awareness, and reduced length of hospital stay. Established patient-related risk factors for SSI, such as obesity, diabetes mellitus, malnutrition, and *S. aureus* colonization are well described [5], [8], [25]. In our cohort, these variables, together with age and sex, remained largely stable over time, suggesting that shifts in patient characteristics were unlikely to account for the observed reduction. Economic analyses further support the implementation of screening and decolonisation protocols, indicating that the cost per infection prevented is substantially lower than the cost of treating PJI [21], [24]. Moreover, recent meta-analyses including more than 56,000 patients undergoing total hip or knee arthroplasty have demonstrated a significant reduction in both overall and *S. aureus*-related PJI risk (p=0.002) [26]. Given the substantial burden associated with *S. aureus* infections – including revision surgery, prolonged hospitalization, and increased healthcare costs – the reduction observed in this study represents a relevant improvement in patient safety. The observed effectiveness may reflect the protocol’s simplicity and the consistent engagement of all stakeholders, including patients, surgeons, physicians, and nursing staff. Limitations of this study include the lack of follow-up screening to confirm successful decolonisation and the non-randomized, observational design with a temporal comparison. Nevertheless, the findings provide real-world evidence supporting the effectiveness of targeted screening and decolonisation. While universal decolonisation may offer logistical advantages and comparable efficacy, particularly in settings with high MSSA prevalence, targeted approaches allow individualized antibiotic prophylaxis and may be preferable in resource-limited settings or where MRSA prevalence is high [22], [27], [28], [29]. In summary, the available evidence suggests that targeted MSSA screening and decolonisation is an effective and economically reasonable strategy to reduce *S. aureus*-associated prosthetic joint infections in elective hip and knee arthroplasty, while universal decolonisation may serve as an alternative in selected settings.

## Conclusion

Our study provides robust real-world evidence that standardized targeted *S. aureus* screening and octenidine-based decolonisation significantly reduce postoperative wound infections in orthopedic patients. It adds valuable data to the growing body of implementation research on infection prevention and supports routine adoption of preoperative decolonisation protocols in high-risk surgical settings.

## Notes

### Competing interests

The authors declare that they have no competing interests.

## Figures and Tables

**Table 1:**
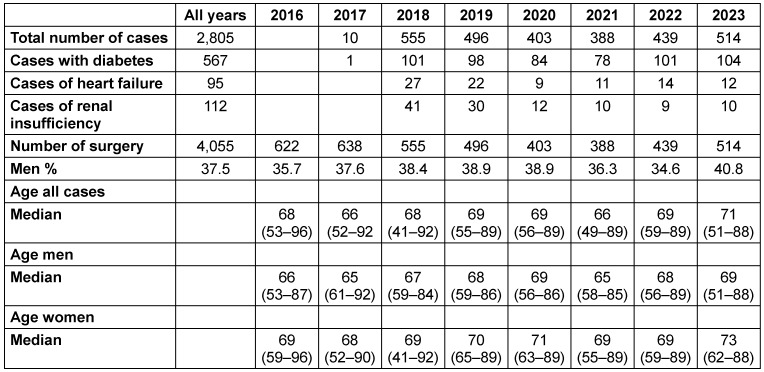
Baseline characteristics of the study population

**Table 2:**
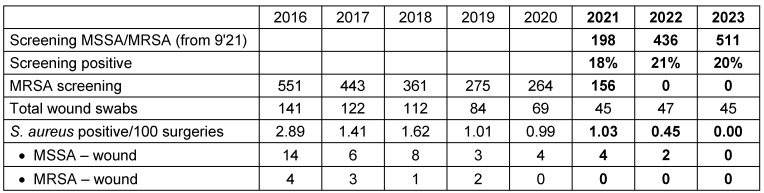
Absolute numbers of decolonisation measures over time
